# TiO_2_/K_2_Ti_6_O_13_ Binary Whiskers Modified Mullite Fiber-Based Materials with Enhanced Thermal Insulation Property

**DOI:** 10.3390/ma19102007

**Published:** 2026-05-12

**Authors:** Xixi Cao, Xueying Zhang, Jiangtao Li, Jiachen Liu

**Affiliations:** Key Lab of Advanced Ceramics and Machining Technology of Ministry of Education, School of Materials Science and Engineering, Tianjin University, Tianjin 300072, China; cxx1087@163.com (X.C.); 1021208011@tju.edu.cn (J.L.)

**Keywords:** mullite fiber, TiO_2_, K_2_Ti_6_O_13_, whisker, infrared shielding

## Abstract

Mullite fiber materials are widely used in high-temperature thermal insulation applications, especially in aerospace thermal protection systems, due to their excellent thermal stability and low thermal conductivity. However, the material exhibits poor resistance to infrared radiative heat transfer at elevated temperatures. Accordingly, a dual-opacifier system composed of TiO_2_ and K_2_Ti_6_O_13_ binary whiskers was proposed as an effective strategy for enhancing thermal insulation performance. MF/TiO_2w_ and MF/TiO_2w_/K_2_Ti_6_O_13w_ were fabricated in this study using a sol–gel method combined with in situ whisker growth. The results show that upright and interlaced K_2_Ti_6_O_13_ and TiO_2_ whiskers were uniformly grown on the fiber surface, contributing to a high infrared reflectance of 97.7% in the wavelength range of 2.5–10 μm. Under a front-side temperature of 1000 °C, the modified mullite fiber-based material exhibits a backside temperature of 177.8 °C, corresponding to a reduction of 71.8 °C compared with the original sample (249.6 °C), demonstrating significantly enhanced thermal insulation performance. In addition, the composite exhibits an ultralow density of less than 0.20 g/cm^3^. The as-prepared thermal insulation material shows a high rebound rate of 76.5% at a strain of 30%, indicating good elasticity. The results demonstrate that the developed composite exhibits excellent infrared shielding and structural stability, confirming that the binary whisker strategy effectively enhances the thermal insulation performance of the mullite fiber-based materials, highlighting its potential for high-temperature aerospace applications.

## 1. Introduction

As important high-temperature thermal insulation materials, ceramic fiber-based materials are widely used in thermal protection systems and aerospace applications, where increasingly stringent requirements have been imposed by the rapid development of hypersonic vehicles and advanced aero-engines, including low density, low thermal conductivity, and excellent thermal and mechanical stability at elevated temperatures [1,2,3,4,5,6,7,8,9,10,11]. As a typical representative of ceramic fiber-based materials, mullite fibers have attracted considerable attention due to their inherent advantages such as low density, low thermal conductivity, resistance to high temperature, and chemical stability [12,13,14,15,16,17,18,19,20,21,22]. Heat transfer in such materials involves conduction, convection, and radiation. However, as the temperature exceeds 800 °C, radiative heat transfer becomes dominant, significantly deteriorating the insulation performance [23,24,25]. Therefore, effectively suppressing infrared radiative heat transfer at high temperatures remains a critical challenge for ceramic fiber-based materials.

The suppression of infrared radiative heat transfer can be achieved by tailoring pore structures, tuning intrinsic optical properties, and constructing hierarchical architectures to enhance infrared attenuation. Among these strategies, the incorporation of infrared shielding agents is considered a promising approach and merits further investigation [26,27,28,29]. The infrared shielding mechanism primarily depends on the optical properties of materials in the infrared region, where increased reflectivity or extinction coefficient reduces radiative heat penetration. Infrared shielding materials can generally be classified into several categories [2,30,31,32]. Zr-based materials, such as ZrO_2_ and ZrSiO_4_, exhibit high infrared reflectivity and excellent chemical stability; however, they suffer from poor dispersion and difficulty in forming effective network structures when used alone [33]. C-based materials, including carbon and SiC, demonstrate excellent infrared shielding performance due to their strong absorption and scattering capabilities. However, they are prone to oxidation under high-temperature aerobic conditions, limiting their application in oxide-based thermal insulation systems [34,35]. Titanium-based materials, such as TiO_2_ and potassium titanate whiskers (e.g., K_2_Ti_6_O_13_), possess good infrared shielding ability. In particular, titanate whiskers with the general formula K_2_Ti_n_O_2n+1_ exhibit high infrared reflectivity and can form interconnected network structures due to their unique one-dimensional morphology [36].

TiO_2_ and potassium titanate whiskers, especially K_2_Ti_6_O_13_, are considered highly effective infrared shielding agents due to their excellent optical properties and high-temperature stability. In addition to intrinsic optical properties, structural factors also play an important role in improving infrared shielding performance, particularly through the introduction of whiskers. The whisker morphology facilitates multiple scattering and the reflection of infrared photons, thereby increasing their propagation path length and enhancing infrared shielding efficiency. TiO_2_ has been widely used in thermal insulation materials, where its incorporation significantly enhances infrared reflectivity and improves insulation performance [37,38,39]. For instance, TiO_2_ nanorod coatings grown on mullite fibers via a seed-assisted hydrothermal method exhibited effective specific extinction coefficients two to four times higher than those of mullite fiber origin in the 3–6 μm range, demonstrating outstanding infrared opacifying capability [39]. Meanwhile, K_2_Ti_6_O_13_ whiskers, typically belonging to the K_2_Ti_n_O_2n+1_ system, possess a high aspect ratio (10–50), enabling the formation of interconnected network structures that increase multiple reflection pathways for infrared radiation [36,40]. In addition, the in situ growth of K_2_Ti_6_O_13_ whiskers on aluminosilicate fibers has been successfully achieved, further demonstrating their feasibility for fiber-based infrared shielding applications [41]. The incorporation of modified K_2_Ti_6_O_13_ whiskers into Al_2_O_3_–SiO_2_ xerogels has been shown to significantly reduce infrared transmittance and thermal conductivity, further confirming their superior infrared shielding and thermal insulation performance.

Conventional approaches typically rely on the external addition of infrared shielding agents, which often leads to poor dispersion and weak interfacial bonding. In this work, TiO_2_ and K_2_CO_3_ are introduced into a mullite fiber matrix, where K_2_Ti_6_O_13_ whiskers are generated in situ during heat treatment, constructing a TiO_2_/K_2_Ti_6_O_13_ dual-opacifier system. The precursors are incorporated via an impregnation method [42,43], followed by heat treatment to achieve in situ whisker growth on and within the fibers, resulting in improved dispersion and interfacial bonding. The resulting whiskers interweave throughout the fiber matrix, forming an interconnected network that increases the tortuosity of heat transfer pathways and enhances interfacial scattering. Meanwhile, the coexistence of TiO_2_ and K_2_Ti_6_O_13_ enables synergistic infrared shielding over a broader wavelength range, leading to enhanced infrared reflectance and suppressed radiative heat transfer at elevated temperatures.

## 2. Materials and Methods

### 2.1. Materials and Experiment Process

Mullite fiber felt (MF, 72 wt% Al_2_O_3_ and 28 wt% SiO_2_, mullite phase) was purchased from Shandong Dongheng Guoxian New Materials Co., Ltd. (Dongying, China), Anatase titanium dioxide (TiO_2_, 99.8%), polyvinyl alcohol (PVA, type 1788, alcoholysis degree 87.0–89.0%), and anhydrous potassium carbonate (K_2_CO_3_, 99%) were obtained from Aladdin Reagent Co., Ltd., Shanghai, China.

**Figure 1:**
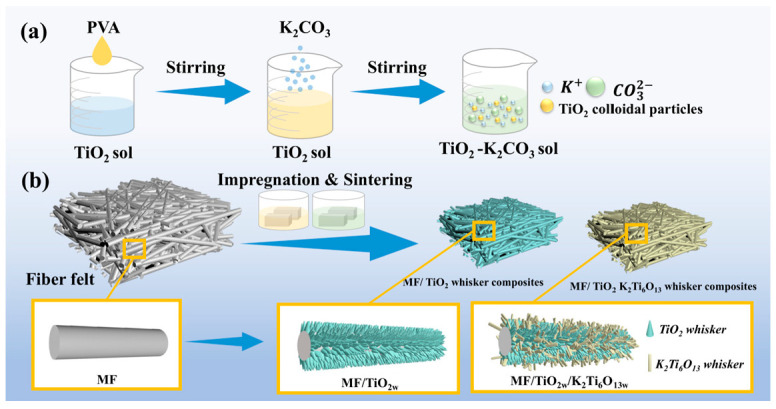
(**a**) The preparation process of sol. (**b**) The preparation process of the MF/TiO_2w_ and MF/TiO_2w_/K_2_Ti_6_O_13w_ composites.

A titanium dioxide sol was prepared. Anatase titanium dioxide (TiO_2_) nanopowder was dispersed in 100 mL of deionized water to obtain suspensions with mass fractions of 1–5 wt%. The mixture was stirred at room temperature for 2 h to ensure uniform dispersion. To further improve the dispersion stability, a pre-prepared 10 wt% polyvinyl alcohol (PVA) aqueous solution was incorporated as a stabilizing agent. The amount of the PVA solution to be added was controlled at 0.5–3 vol% relative to the TiO_2_ sol. The mixture was further stirred to obtain a uniform and stable milky suspension. Subsequently, anhydrous potassium carbonate was added according to molar ratios of K_2_CO_3_ to TiO_2_ of 1:5, 1:5.5, 1:6, 1:6.5, and 1:7 (denoted as K:T hereafter). The total solid content of the system was then adjusted to 10 wt%, and a homogeneousTiO_2_-K_2_CO_3_ composite sol was obtained after stirring. The specific preparation process is shown in Figure 1a.

The prepared sol was applied onto the mullite fiber felt (MF) via a sol–gel impregnation process. MF samples (50 mm × 50 mm × 10 mm) were impregnated with a sol/felt volume ratio of ~2:1 to ensure complete infiltration. The MF matrix was initially subjected to heating at 600 °C for 2 h in a high-temperature furnace to effectively eliminate organic contaminants from the surface. Subsequently, the MF preform underwent sequential TiO_2_ sol impregnation, gelation, and thermal treatment as outlined: (1) Vacuum Impregnation: the pretreatment MF matrix was submerged in the prepared sol and subjected to a vacuum for 10 min to guarantee complete infiltration of the sol into the fibers. (2) Gelation: the impregnated preform underwent drying in an oven at 60 °C to finalize the sol–gel transition. (3) Heat Treatment: the gelled composite green body was subjected to heat treatment at 800 °C and 1000 °C for 1 h, yielding TiO_2_-coated MF samples, denoted as MF/TiO_2w_-800 and MF/TiO_2w_-1000, respectively. The detailed preparation procedure is illustrated in Figure 1b.

Based on the above procedure, the TiO_2_-K_2_CO_3_ composite sol was further impregnated into MF, followed by heat treatment at 1100 °C for 2 h to obtain the final composite, denoted as MF/TiO_2w_/K_2_Ti_6_O_13w_.

### 2.2. Characterization

Scanning electron microscopy (SEM) (S-4800 scanning electron microscope, Hitachi, Tokyo, Japan) and energy dispersive spectroscopy (EDS) were selected for analyzing the surface and cross-sectional morphology, and the elemental distribution of the MF/TiO_2w_ and MF/TiO_2w_/K_2_Ti_6_O_13w_ composites. For SEM and EDS characterization, fiber samples were carefully taken from the fibrous felts using tweezers, mounted on conductive copper stubs, and sputter-coated with gold for 100 s to enhance electrical conductivity prior to testing. Observations were then carried out under an accelerating voltage of 15 kV.

The crystal phases of the mullite fiber, MF/TiO_2w_, and MF/TiO_2w_/K_2_Ti_6_O_13w_ composites were characterized by X-ray diffraction spectroscopy (XRD) (D/MAX-2500 X-ray diffractometer, Rigaku, Tokyo, Japan). The 2θ range was from 10° to 90°. Infrared reflectance spectra were tested using a fully vacuum infrared spectrometer (VERTEX 80v, Bruker, Ettlingen, Germany) over the wavenumber range of 400–2000 cm^−1^. Compression-recovery behavior analyses were carried out using an electronic universal testing machine (CMT4303, Meister Industrial Systems, Jinan, China) with a preload of 0.05 N, a compression rate of 3 mm/min, and a compression deformation of 30% of the sample thickness for five loading–unloading cycles. The resilience ratio and maximum compressive stress of the fiber felts were determined from the stress–strain curves. Real-time back-surface temperature evolution was monitored using an infrared thermal imaging system (LT7-P, Zhejiang Dali Technology Co., Ltd., Hangzhou, China). The bulk density of the samples was calculated from their measured geometric volume and mass.

## 3. Results

### 3.1. Morphology and Microstructure

**Figure 2:**
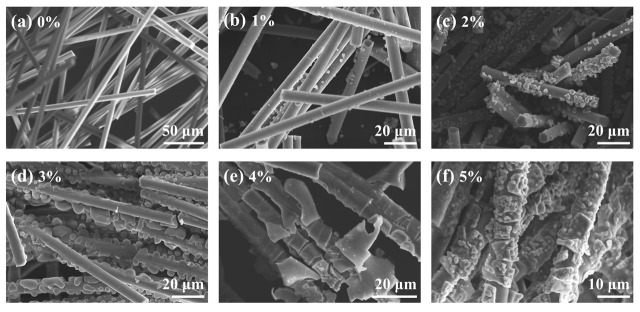
SEM images of fibers impregnated with different concentrations of TiO_2_ sol after 800 °C heat treatment: (**a**) 0%, (**b**) 1%, (**c**) 2%, (**d**) 3%, (**e**) 4%, (**f**) 5%.

We examined the impact of TiO_2_ sol concentration on the coating morphology of mullite fibers. Figure 2 illustrates the SEM morphologies of mullite fibers infused with different concentrations of TiO_2_ sol after heat treatment at 800 °C. As the concentration of TiO_2_ sol escalates from 0% to 3%, the quantity of particles deposited on the fiber surface significantly increases, resulting in a progressive enhancement in coating coverage. At a sol concentration of 3%, particle deposition approaches saturation; raising the concentration to 4% and 5% causes significant particle aggregation and clumping, potentially resulting in localized cracking and delamination of the coating layer. Furthermore, TiO_2_ particles adhere to the fiber surface in an agglomerated state across all concentrations, failing to form a continuous and uniform coating layer. This indicates that basic sol impregnation is inadequate for achieving optimal particle dispersion, hence requiring the incorporation of PVA as a dispersant to improve particle distribution and coating uniformity.

**Figure 3:**
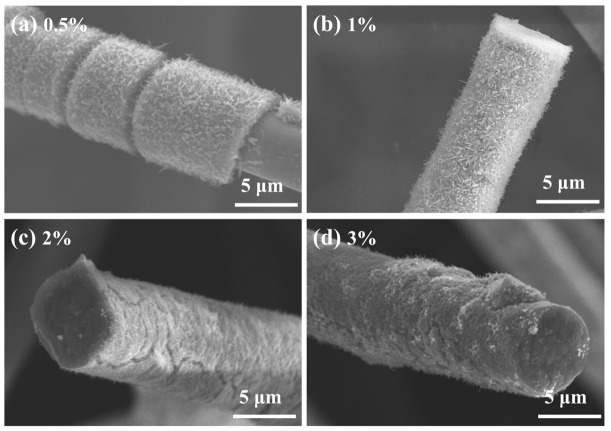
SEM images of fibers impregnated with different concentrations of PVA after heat treatment at 800 °C: (**a**) 0.5%, (**b**) 1%, (**c**) 2%, (**d**) 3%.

The effect of PVA addition on the morphology of fiber coatings was then investigated. The PVA addition denotes a volume proportion of 10 wt% PVA solution added to 100 mL of impregnation solution (e.g., 0.5 mL equates to 0.5%). Figure 3 illustrates the SEM morphologies of samples infused with different PVA concentrations following thermal treatment at 800 °C. The incorporation of PVA markedly enhanced the dispersion of TiO_2_ particles and the homogeneity of the coating, resulting in nearly complete coverage of the fiber surface. At a PVA concentration of 0.5%, particle deposition occurred; however, substantial regions of the fiber surface remained uncoated. With the increase to 1%, the fiber surface was uniformly covered with a layer of short, dense whiskers, indicating the optimal coating condition. Increasing the addition to 2% and 3% led to local particle aggregation and coating layer inhomogeneity, with no significant morphological difference between the two, suggesting that the coating had attained saturation.

**Figure 4:**
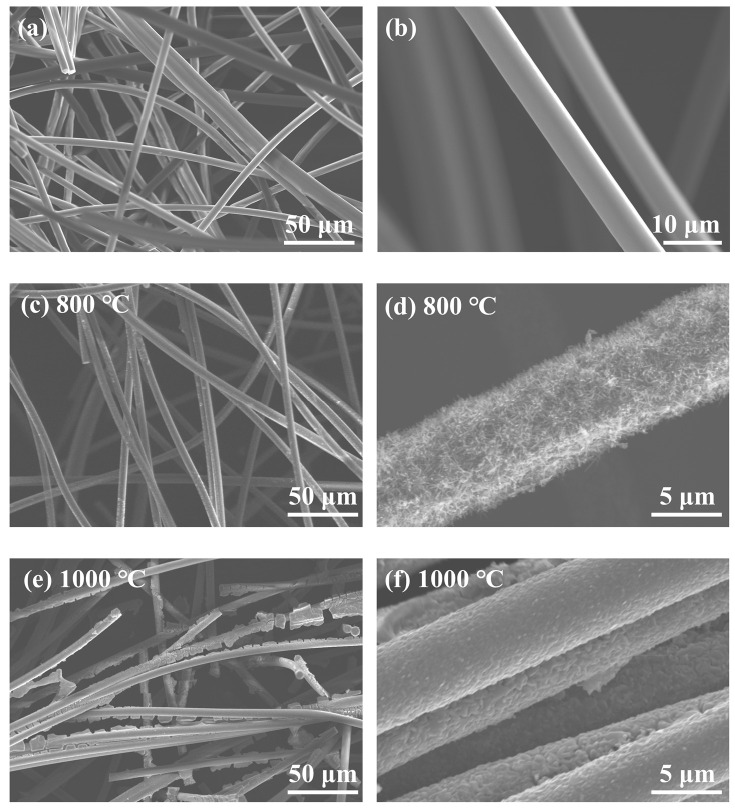
SEM images of MF and MF/TiO_2w_ at different heat treatment temperatures: (**a**,**b**) MF, (**c**,**d**) 800 °C, (**e**,**f**) 1000 °C.

The impact of heat treatment temperature on the shape of MF/TiO_2w_ fibers was further examined. Figure 4a,b depict the untreated control sample, wherein the pristine mullite fibers have a smooth surface and a randomly interwoven network structure characterized by a loose arrangement, devoid of any discernible secondary phase, thereby entirely preserving the initial morphological attributes of the mullite fibers. Following heat treatment at 800 °C (Figure 4c,d), the fibers preserve a continuous interlocking configuration with an unblemished overall structure in the low-magnification perspective. At high magnification, a dense, whisker-like structure is evenly developed on the surface of the mullite fiber, characterized by fine dimensions and consistent distribution, demonstrating the successful in situ development and deposition of target whiskers on the mullite fiber surface at this temperature. Upon elevating the treatment temperature to 1000 °C (Figure 4e,f), the whisker structure on the fiber surface vanishes, and the initial fine morphology evolves into a continuous and coarse coating layer, leading to a marked increase in surface roughness. Simultaneously, certain fibers exhibit morphological degradation and structural fractures, indicating that elevated temperatures result in sintering and recrystallization of the whiskers, compromising their original structure and undermining the integrity of the mullite fiber substrate. A thorough comparison indicates that 800 °C is the ideal temperature for fabricating whisker-modified composite fibers on the mullite fiber surface, resulting in a composite system characterized by uniform whisker distribution and an intact fiber structure. A temperature of 1000 °C results in the disintegration of the whisker structure and fiber deterioration, adversely affecting the preservation of the whisker morphology.

**Figure 5:**
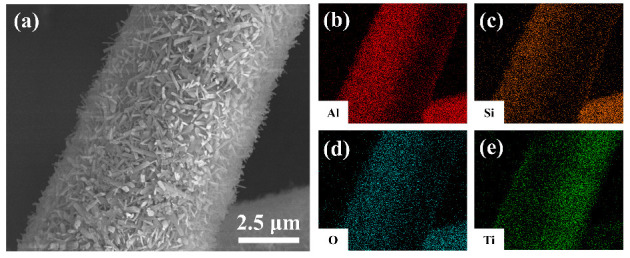
(**a**) SEM and EDS analysis of MF/TiO_2w_ at 800 °C: (**b**) Al, (**c**) Si, (**d**) O, (**e**) Ti.

Figure 5 illustrates the SEM morphology and EDS elemental mapping analysis of the MF/TiO_2_ sample subsequent to heat treatment at 800 °C. Figure 5a shows that the surface of the mullite fiber is entirely enveloped by a dense layer of short, randomly oriented whisker-like structures, resulting in a rough hierarchical architecture that aligns with prior observations. The EDS elemental mapping results (Figure 5b–e) further validate the elemental composition: the signals for Al and Si correspond precisely to the mullite fiber matrix, O is extensively distributed throughout both the fiber and whisker regions, while Ti is specifically concentrated in the whisker layer on the fiber surface. This clearly demonstrates that Ti has been effectively included to create a TiO_2_-based whisker coating, with all elements uniformly distributed in the composite system, exhibiting no obvious segregation.

**Figure 6:**
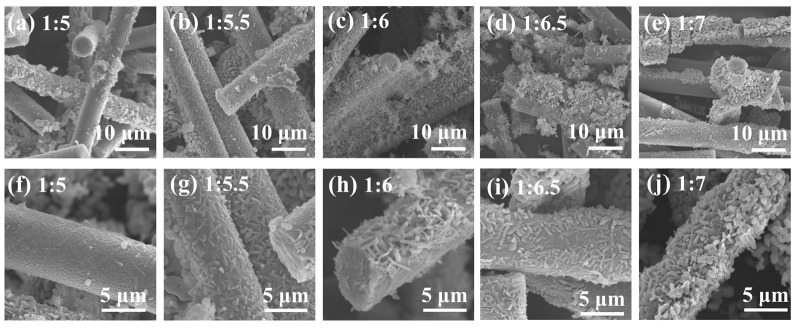
SEM images of MF/TiO_2w_/K_2_Ti_6_O_13w_ whisker composites under different K:T ratios: (**a**,**f**) 1:5, (**b**,**g**) 1:5.5, (**c**,**h**) 1:6, (**d**,**i**) 1:6.5, (**e**,**j**) 1:7.

Figure 6 illustrates the SEM morphologies of MF/TiO_2w_/K_2_Ti_6_O_13w_ whisker composites synthesized with varying K:T ratios. As the K:T ratio progressively increases from 1:5 to 1:7, signifying a gradual reduction in the relative concentration of potassium carbonate, the coating morphology on the fiber surface undergoes substantial alteration. At a K:T of 1:5, minimal sparse particle products stick to the fiber surface, leading to inadequate whisker growth and the absence of a continuous coating layer, which results in suboptimal coverage. After increasing the K:T to 1:5.5, whisker-like structures emerge on the fiber surface; however, the majority remain flattened against the fibers with a dispersed distribution, and the coating density remains inadequate. Increasing the K:T to 1:6 optimally enhances whisker growth: the fiber surface is entirely enveloped by numerous short, upright, interlaced whiskers exhibiting varied orientations and uniform distribution, resulting in dense coverage and robust bonding, exemplifying the ideal morphology. As the K:T escalates to 1:6.5, excessive whisker proliferation transpires, resulting in the majority of whiskers becoming flattened and affixed to the fiber surface, exhibiting disorderly arrangement and localized particle aggregation, hence diminishing coating uniformity. When the K:T is overly elevated at 1:7, an inadequate potassium source results in significant whisker aggregation and clumping, as well as localized coating breaking and substantial buildup, which damages portions of the fiber structure and leads to markedly degraded morphology. A thorough analysis reveals that K:T = 1:6 is best for synthesizing high-quality MF/TiO_2w_/K_2_Ti_6_O_13w_ composites, resulting in a composite coating characterized by upright and interlaced whiskers, uniform distribution, and an unbroken fiber structure.

**Figure 7:**
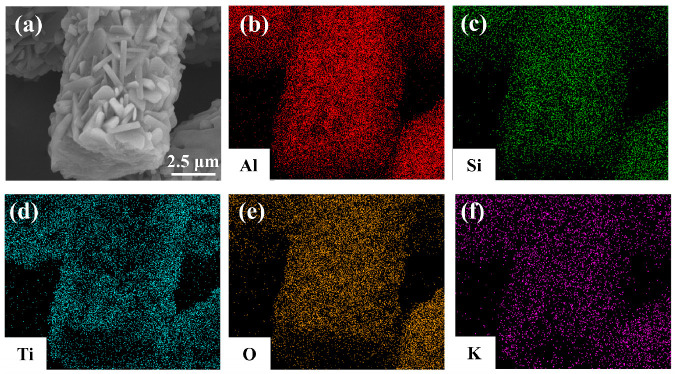
(**a**) SEM and EDS analysis of MF/TiO_2w_/K_2_Ti_6_O_13w_ composites: (**b**) Al, (**c**) Si, (**d**) Ti, (**e**) O, (**f**) K.

Figure 7 illustrates the SEM morphology and EDS elemental mapping study of the MF/TiO_2w_/K_2_Ti_6_O_13w_ whisker composite. Figure 7a shows that the surface of the mullite fiber is entirely enveloped by numerous dense, whisker-like structures exhibiting regular shapes and homogeneous distribution, aligning with the previously observed ideal morphological qualities. The EDS elemental mapping results (Figure 7b–f) corroborate the elemental composition: the signals for Al and Si correspond exactly to the mullite fiber matrix, whereas Ti and K are uniformly concentrated in the whisker coating layer on the fiber surface, and O is extensively distributed throughout both the matrix and whisker regions. All components are evenly distributed inside the composite system, exhibiting no discernible segregation. This signifies that Ti- and K-containing whiskers have been effectively produced in situ and uniformly deposited on the surface of the mullite fiber. Subsequent XRD analysis further confirms that the composite system has both TiO_2_ and K_2_Ti_6_O_13_ phases.

### 3.2. Evolution of Whisker Phase Composition

**Figure 8:**
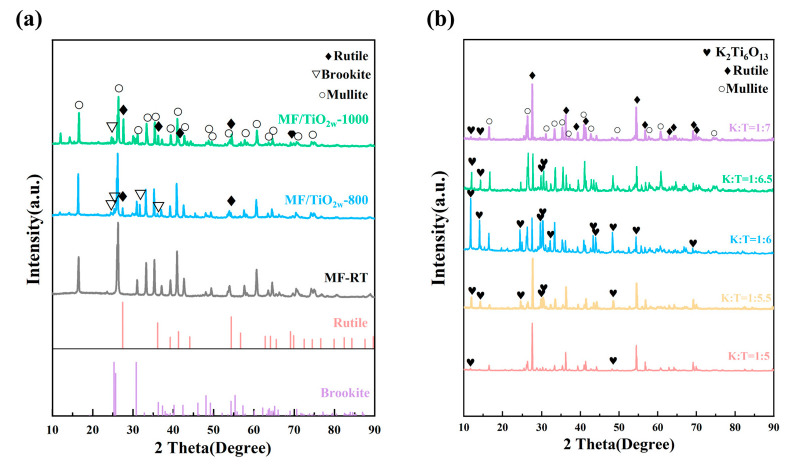
(**a**) XRD patterns of MF/TiO_2w_ after heat treatment at different temperatures; (**b**) XRD patterns of MF/TiO_2w_/K_2_Ti_6_O_13w_ whisker composites under different K:T ratios.

The XRD patterns of different materials are shown in Figure 8. Figure 8a presents the phase compositions of origin MF, MF/TiO_2w_-800, and MF/TiO_2w_-1000. The diffraction peaks of the pristine MF sample can be well indexed to mullite (PDF#98-000-0319), with no detectable impurity peaks, indicating high phase purity.

After heat treatment at 800 °C, additional diffraction peaks corresponding to brookite TiO_2_ (PDF#98-000-0128) and rutile TiO_2_(PDF#98-000-0375) are observed, confirming the successful deposition of TiO_2_ on the fiber surface. When the heat treatment temperature increases to 1000 °C, the diffraction peaks of brookite in MF/TiO_2w_-1000 become significantly weaker, while the rutile peaks are noticeably enhanced. This result indicates the gradual phase transformation of brookite into the thermodynamically more stable rutile phase at elevated temperature.

Figure 8b shows the phase compositions of MF/TiO_2w_/K_2_Ti_6_O_13w_ composites prepared with different K:T ratio. All samples display distinct diffraction peaks of K_2_Ti_6_O_13_(PDF#01-074-0275) and rutile TiO_2_. As the K:T ratio increases from 1:5 to 1:7, the characteristic peak intensity of K_2_Ti_6_O_13_ first increases and then decreases, reaching its maximum at K:T = 1:6, where the diffraction peaks of K_2_Ti_6_O_13_ are also the most prominent among all detected phases. In comparison, relatively weaker K_2_Ti_6_O_13_ diffraction peaks are observed at the other K:T ratios.

### 3.3. Infrared Shielding and Thermal Insulation Performance

**Figure 9:**
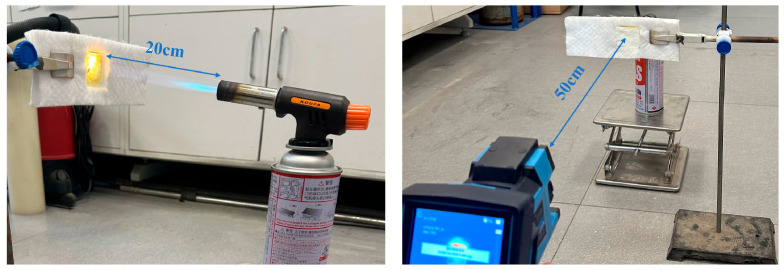
Experimental setup diagram for recording infrared thermal imaging of the sample using a thermal infrared image under a butane blow torch.

The infrared radiation shielding capability and high-temperature thermal insulation performance of the fiber felts were evaluated using a backside temperature test setup, as illustrated in Figure 9. In this test, a butane torch was used to continuously heat the front surface of the sample from a distance of 20 cm to simulate a high-temperature environment, while an infrared thermal imaging camera monitored the backside temperature in real time from a distance of 50 cm. Due to the open-flame heating conditions, the front-side temperature may exhibit slight fluctuations caused by ambient airflow disturbances. Nevertheless, all samples were subjected to comparable thermal conditions, ensuring the reliability of performance comparison.

**Figure 10:**
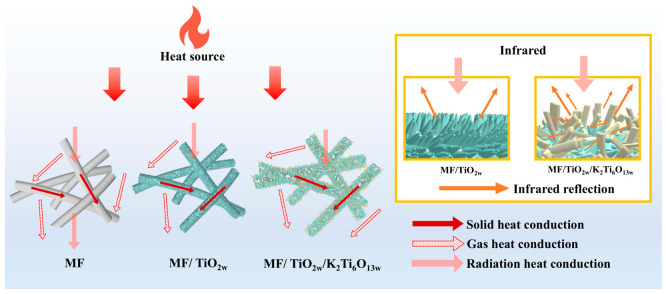
Schematic illustration of the thermal insulation mechanism of MF/TiO_2w_/K_2_Ti_6_O_13w_ composite fiber felts.

The thermal insulation mechanism of the MF/TiO_2w_/K_2_Ti_6_O_13w_ composite fiber felts is schematically illustrated in Figure 10, showing the primary heat transfer modes in porous fibrous materials and the suppressing effect of whisker modification on heat transfer.

In pristine mullite fibers, heat is transferred to the cold side through solid conduction along the fiber framework, gas conduction within the pores, and radiative transfer at elevated temperatures, which limits the thermal insulation performance. After the introduction of TiO_2_/K_2_Ti_6_O_13_ whiskers, a dense whisker layer is formed on the fiber surface, significantly enhancing the reflection of infrared radiation, thereby effectively suppressing radiative heat transfer at high temperatures. Meanwhile, the whiskers grow in an interlaced manner on the fiber surface and within the pores, forming a partially interconnected three-dimensional network structure. This structure increases the tortuosity of the heat transfer pathways and hinders heat propagation through the material.

In addition, the whiskers exhibit good interfacial bonding with the fiber matrix, helping to maintain structural stability and further suppress heat transfer. As a result of these multi-scale structural synergistic effects, the composite fiber felts exhibit significantly improved thermal insulation performance at elevated temperatures.

**Figure 11:**
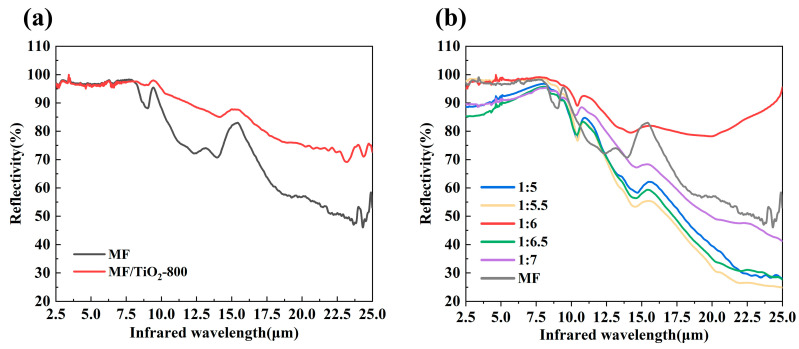
Infrared reflectance spectra of different samples: (**a**) MF/TiO_2w_ at different heat treatment temperatures; (**b**) MF/TiO_2w_/K_2_Ti_6_O_13w_ at different K:T ratios.

Figure 11 provides a detailed study of the infrared reflectance spectra, indicating that all changed samples display enhanced infrared shielding efficacy relative to MF. The MF/TiO_2w_-800 sample exhibits a reflectance exceeding 70% in the mid- to far-infrared range (8–25 μm), markedly surpassing MF, which registers below 50% in the identical spectral region. This verifies that the TiO_2_ whisker coating produced at 800 °C efficiently reflects and scatters infrared energy and already shows competitive performance in the 2.5–3 μm range compared with previously reported systems [44,45]. Among the MF/TiO_2w_/K_2_Ti_6_O_13w_ composites with varying K:T ratios, the sample exhibiting K:T = 1:6 demonstrated the best performance, attaining an average reflectance of 97.7% in the 2.5–10 μm range and maintaining stability at approximately 80% in the extended wavelength region of 10–25 μm, significantly outperforming samples with alternative ratios and also showing improved performance compared with other reported binary coating systems [46], relevant detailed data are shown in Table S1. The infrared reflectance progressively diminishes when K:T diverges from 1:6. This trend is consistent with the microstructural observations. At K:T = 1:6, well-developed and uniformly distributed K_2_Ti_6_O_13_ whiskers are formed, leading to an effective multi-scale structure for infrared reflection and enhanced attenuation of infrared radiation. In contrast, non-optimal ratios tend to cause abnormal whisker growth or aggregation, which weakens the shielding performance.

The spectral results are consistent with the microstructures observed by SEM and XRD, as well as the thermal insulation performance reflected in the backside temperature measurements. Overall, the results indicate that controlling the microstructure through composition and heat treatment is key to improving infrared shielding performance.

**Figure 12:**
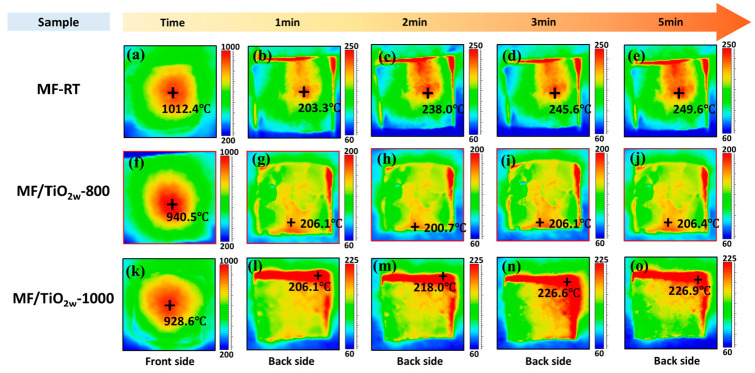
Infrared thermography images of samples recorded under a butane torch. (**a**–**e**) MF; (**f**–**j**) MF/TiO_2w_-800; (**k**–**o**) MF/TiO_2w_-1000. Each group shows front side and back side at 1, 2, 3, and 5 min.

Figure 12 shows the temporal evolution of the backside temperature of the samples over 5 min under sustained high-temperature heat flow, evaluating their thermal insulation and infrared shielding performance. The front-surface flame conditions were kept constant, and a stable flame (~1000 °C) was maintained prior to measurement to ensure thermal stabilization. Under these identical conditions, the front-surface temperature exhibits a decreasing trend with the introduction and variation in the modification, indicating that the surface thermal response is also affected by the modified infrared radiative properties of the material under the same heat flux [47,48].

Due to structural limitations of the test setup, flame infiltration may occur at the sample–fixture interface, leading to locally elevated temperatures at the edges. Therefore, only the backside temperature in the central region is considered for analysis.

As the heating duration increased from 1 min to 5 min, the backside temperature of all samples gradually rose and stabilized. The comparison of various samples indicates that the backside temperatures of the TiO_2_ whisker-modified samples are markedly lower than MF, demonstrating enhanced thermal insulation efficacy. The MF/TiO_2w_-800 sample, subjected to heat treatment at 800 °C, demonstrated superior performance: after 5 min of heating, the maximum backside temperature reached only 206.4 °C, with the majority of areas remaining below 200 °C, significantly lower than that of other samples. This outcome aligns closely with prior SEM and XRD analyses: the uniform whisker coating and the amalgamation of TiO_2_ crystalline phases at 800 °C create an efficient infrared shielding and heat conduction barrier structure, markedly improving the high-temperature thermal insulation capacity of the fiber felts, thereby validating the efficacy of the whisker modification strategy in enhancing thermal insulation performance.

**Figure 13:**
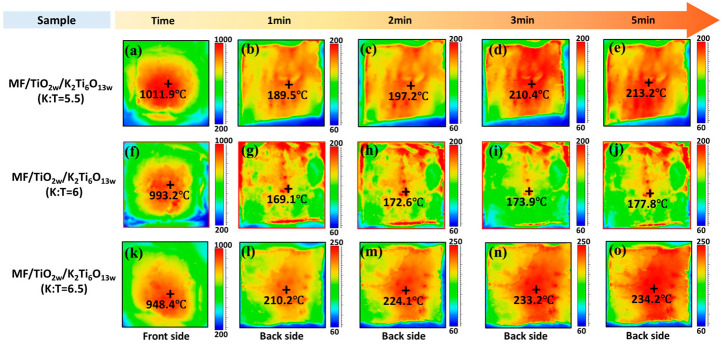
Infrared thermography images of samples recorded under a butane torch. (**a**–**e**) MF/TiO_2w_/K_2_Ti_6_O_13w_ (K:T = 1:5.5); (**f**–**j**) MF/TiO_2w_/K_2_Ti_6_O_13w_ (K:T = 1:6); (**k**–**o**) MF/TiO_2w_/K_2_Ti_6_O_13w_ (K:T = 1:6.5). Each group shows front side and back side at 1, 2, 3, and 5 min.

Figure 13 illustrates the temperature progression on the reverse side of MF/TiO_2w_/K_2_Ti_6_O_13w_ composite samples with varying K:T ratios over a 5 min duration under sustained high-temperature heat flux, with the objective of assessing the influence of whisker phase modulation on thermal insulation efficacy. The test conditions align with the prior analysis: the core front-side temperature of all composite samples surpasses 800 °C, with localized areas above 1000 °C, imitating an extreme high-temperature service environment. The structural constraints of the test apparatus result in flame penetration through the space between the sample and fixture, leading to abnormally elevated temperatures at the edges; therefore, only the middle region is examined. As the heating duration extends from 1 min to 5 min, the temperature of the rear of each composite sample progressively rises and stabilizes. The comparison of various K:T ratios indicates that the K:T = 5.5 sample reaches a maximum backside temperature of 213.2 °C after 5 min of heating, demonstrating moderate thermal insulation performance. In contrast, the K:T = 1:6 sample exhibits superior performance, with a maximum backside temperature of only 177.8 °C after the same duration, significantly lower than other ratios, and a more uniform temperature distribution across the sample. However, when the K:T increases to 1:6.5, the maximum backside temperature escalates to 234.2 °C, signifying a decline in thermal insulation efficacy. This trend aligns well with the SEM and XRD analyses: at K:T = 1:6, upright, interlaced, and uniformly distributed K_2_Ti_6_O_13_ whiskers are generated on the fiber surface, and the crystallinity of the composite phase is maximized. This structure can efficiently diminish infrared radiation by interface reflection and scattering, while enhancing the tortuosity of heat transfer pathways, thus optimally suppressing heat transfer through the material. Conversely, inadequate or excessive K:T results in insufficient whisker development or significant agglomeration, leading to diminished infrared shielding and thermal insulation efficacy.

### 3.4. Physical and Mechanical Properties

**Figure 14:**
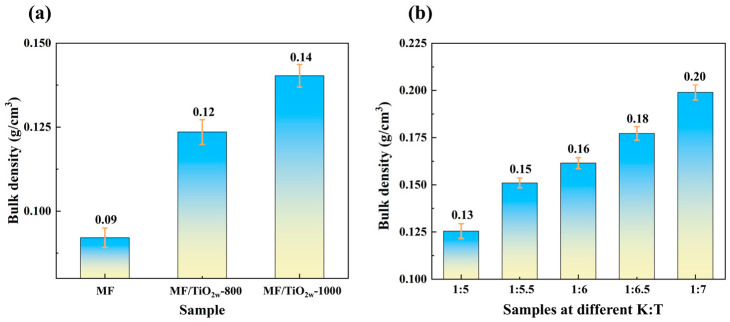
Bulk density plots of different samples: (**a**) density variation in MF/TiO_2w_ at different heat treatment temperatures; (**b**) density variation in MF/TiO_2w_/K_2_Ti_6_O_13w_ at different K:T ratios.

Figure 14 shows the bulk density of different samples, reflecting the density change in fiber felts prior to and after modification. In Figure 14a, the bulk density of the MF sample is 0.09 g/cm^3^. Following the incorporation of TiO_2_ whiskers, the densities of MF/TiO_2w_-800 and MF/TiO_2w_-1000 samples increase to 0.12 g/cm^3^ and 0.14 g/cm^3^, respectively. This phenomenon is attributed to the in situ growth of TiO_2_ whiskers on the fiber surface, which fills part of the pores, resulting in an augmented sample mass, thus enhancing the density. Simultaneously, heat treatment at 1000 °C further promotes fiber sintering and densification, further increasing the density. Figure 14b indicates that the bulk density of MF/TiO_2w_/K_2_Ti_6_O_13w_ composite samples exhibits a consistent increase with the rise in K:T, rising from 0.13 g/cm^3^ at K:T = 1:5 to 0.20 g/cm^3^ at K:T = 1:7. The rise in relative TiO_2_ concentration leads to the generation and accumulation of more whiskers on the fiber surface, which fill additional pores and progressively enhances the densification degree of the samples. The whisker composite modification increases the density of fiber felts to a degree, yet it remains low (≤0.20 g/cm^3^), meaning it can be classified as a lightweight thermal insulation material, thereby offering a structural foundation for subsequent high-temperature thermal insulation applications.

**Figure 15:**
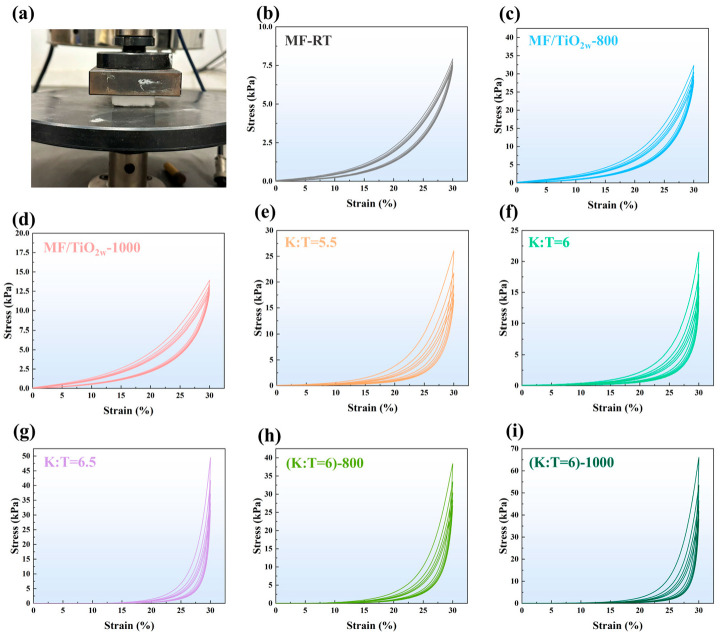
(**a**) Compression resilience device schematic; stress–strain curves of different samples: (**b**) MF-RT; (**c**) MF/TiO_2w_-800; (**d**) MF/TiO_2w_-1000; (**e**) MF/TiO_2w_/K_2_Ti_6_O_13w_ (K:T = 1:5.5); (**f**) MF/TiO_2w_/K_2_Ti_6_O_13w_ (K:T = 1:6); (**g**) MF/TiO_2w_/K_2_Ti_6_O_13w_ (K:T = 1:6.5); (**h**) MF/TiO_2w_/K_2_Ti_6_O_13w_ (K:T = 1:6)-800; (**i**) MF/TiO_2w_/K_2_Ti_6_O_13w_ (K:T = 1:6)-1000.

**Figure 16:**
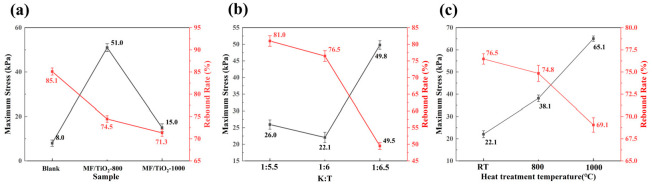
Maximum stress and rebound rates of different samples: (**a**) MF and MF/TiO_2w_ at different heat treatment temperatures; (**b**) MF/TiO_2w_/K_2_Ti_6_O_13w_ at different K:T ratios. (**c**) MF/TiO_2w_/K_2_Ti_6_O_13w_ (K:T = 1:6) at different heat treatment temperatures.

Figure 15 and Figure 16 thoroughly illustrate the compressive mechanical properties, rebound characteristics, and high-temperature thermal stability of several samples. The MF sample exhibits elastic–plastic deformation behavior typical of lightweight porous fiber felts, with a maximum compressive stress of only 8.0 kPa and a rebound ratio of 85.1%, reflecting its basic mechanical performance and good elastic recovery. After modification with TiO_2_ whiskers, the maximum stress of the MF/TiO_2w_-800 sample increases significantly to 51.0 kPa, while the rebound rate remains at 74.5%. This is attributed to the uniform whiskers grown in situ at 800 °C, which form a reinforcing skeleton on the fiber surface, enhancing compressive strength without severely damaging the porous elastic structure of the fiber felt. In contrast, the MF/TiO_2w_-1000 sample shows reduced mechanical performance and recovery, with a maximum stress of 15.0 kPa and a rebound ratio of 71.3%, due to fiber embrittlement caused by high-temperature sintering.

For the MF/TiO_2w_/K_2_Ti_6_O_13w_ composite samples, the mechanical properties vary regularly with the K:T ratio: the maximum stress first decreases to 22.1 kPa at K:T = 1:6 and then increases to 49.8 kPa at K:T = 6.5, while the rebound ratio continuously drops from 80.5% to 49.5%. This is because the introduction of whiskers increases the sample density and solid-phase contact, enhancing compressive strength but sacrificing some elasticity. Among them, the K:T = 1:6 sample achieves the best balance between mechanical strength and rebound ratio, which is highly consistent with its optimal thermal insulation performance.

To evaluate the thermo-mechanical stability, the K:T = 1:6 sample was examined after exposure to different temperatures, including room temperature, 800 °C, and 1000 °C. With increasing heat-treatment temperature, the maximum compressive stress increases significantly from 22.1 kPa to 65.1 kPa, while the rebound ratio only decreases slightly from 76.5% to 69.1%, retaining approximately 70% of its elastic recovery capability. Notably, even after exposure to 1000 °C, the sample still maintains relatively high mechanical strength and recovery performance, indicating good structural stability under severe thermal conditions. This behavior is attributed to the further densification of the whisker–fiber network at elevated temperatures, which enhances load-bearing capacity without inducing significant brittle fracture or excessive loss of elasticity, demonstrating excellent thermo-mechanical stability and strong potential for high-temperature applications. 

In addition, unlike conventional systems that rely on a single infrared shielding component, the present work employs a dual-whisker strategy combining TiO_2_ and K_2_Ti_6_O_13_. This synergistic configuration contributes to improved infrared attenuation and thermal insulation performance, demonstrating the advantage of the proposed design.

## 4. Conclusions

In this study, mullite fiber-based composites (MF/TiO_2w_ and MF/TiO_2w_/K_2_Ti_6_O_13w_) were fabricated via a sol–gel method combined with in situ whisker growth, and the effects of heat-treatment temperature and K:T ratio on the thermal and mechanical properties were investigated. Heat treatment at 800 °C led to the formation of uniformly distributed TiO_2_ whiskers, enhancing infrared reflectivity and reducing the backside temperature to 206.4 °C after 5 min of flame exposure, whereas treatment at 1000 °C caused fiber sintering and performance deterioration. At a K:T ratio of 1:6, the introduction of K_2_Ti_6_O_13_ whiskers increased the infrared reflectivity to 97.7% (2.5–10 μm) and reduced the backside temperature from 250 °C to 177.8 °C. Deviation from this ratio weakened the infrared shielding effect due to insufficient growth or agglomeration of whiskers. Meanwhile, the sample exhibited a maximum compressive stress of 22.1 kPa and a rebound ratio of 76.5%. After exposure to 1000 °C, the sample maintained a high rebound ratio reaching 69.1%, demonstrating excellent thermo-mechanical stability. These results demonstrate the superiority of the synergistic binary whisker design over previously reported coating systems. Overall, the synergistic effect of TiO_2_ and K_2_Ti_6_O_13_ whiskers enables simultaneous improvement in thermal insulation and mechanical performance of the mullite-based materials, indicating strong potential for high-temperature applications.

## Data Availability

The original contributions presented in this study are included in the article/Supplementary Material. Further inquiries can be directed to the corresponding authors.

## Supplementary Materials

The following supporting information can be downloaded at: https://www.mdpi.com/article/10.3390/ma19102007/s1, Table S1: Comparison of infrared reflectance properties of different coating systems.
