# Supplementary material for: TiO2/K2Ti6O13 Binary Whiskers Modified Mullite Fiber-Based Materials with Enhanced Thermal Insulation Property
Source: Materials (Basel). 2026 May 12;19(10):2007. doi: 10.3390/ma19102007 (PMC13208818; doi:10.3390/ma19102007)
Supplement: Supplementary file 1 [file materials-19-02007-s001.zip › materials-4284231-supplementary.pdf]

**Table S1.** Comparison of infrared reflectance properties of different coating systems

| Sample/System | Matrix | Reflective wavelength range | Reflectance |
|---|---|---|---|
| $TiO_2$ coating | $ZrO_2$ fiber | 2.5–3 μm | <95% |
| Potassium hexatitanate coating | Corundum plate | 2.5–3 μm | <80% |
| $TiO_2$ coating | Flexible alumina fiber fabric | 2.5–3 μm | <60% |
| | | 3–14μm | <60% |
| $ZrO_2/SiC_w$ coating | | 2.5–3 μm | <40% |
| | | 3–14μm | <40% |
| This work($TiO_2$ whiskers coating) | Mullite fiber | 2.5–3 μm | 97% |
| | | 3–14μm | 94% |
| This work($TiO_2/K_2Ti_6O_{13}$ dual whiskers coating) | | 2.5–3 μm | 97% |
| | | 3–14μm | 95% |
